# Comparative study of triple intravenous chemotherapy versus dual chemotherapy combined with hepatic arterial infusion in patients with liver metastases from colorectal cancer

**DOI:** 10.3389/fonc.2026.1794578

**Published:** 2026-06-18

**Authors:** Aimin Zhou, Lichao Wang, Chijin Xiao, Yunan Shi

**Affiliations:** Department of Medical Oncology, Yichun People's Hospital, Yichun, Jiangxi, China

**Keywords:** colorectal liver metastases, FOLFIRI, FOLFOXIRI, hepatic arterial infusion chemotherapy, progression-free survival

## Abstract

**Objective:**

This study aimed to compare the therapeutic efficacy and safety of FOLFOXIRI triplet chemotherapy with FOLFIRI combined with hepatic arterial infusion chemotherapy (HAIC) in patients with unresectable colorectal liver metastases.

**Methods:**

This was a single-center retrospective cohort study conducted at the Department of Medical Oncology, The Affiliated Hospital of Yichun University. A total of 132 patients with unresectable colorectal liver metastases treated between January 2019 and December 2023 were included. Patients were divided into the FOLFOXIRI group (n = 67) and the FOLFIRI plus HAIC group (n = 65). The primary endpoint was progression-free survival (PFS). Secondary endpoints included overall survival (OS), objective response rate (ORR), disease control rate (DCR), conversion-to-resection outcomes, and adverse events. Survival outcomes were evaluated using Kaplan–Meier analysis and Cox proportional hazards regression.

**Results:**

The FOLFIRI plus HAIC group showed a higher ORR than the FOLFOXIRI group [37/65 (56.9%) vs. 30/67 (44.8%), p = 0.163] and a higher DCR [58/65 (89.2%) vs. 46/67 (68.7%), p = 0.004]. Kaplan–Meier analysis demonstrated significantly longer PFS in the FOLFIRI plus HAIC group than in the FOLFOXIRI group (10.3 vs. 8.2 months; log-rank p = 0.038). In the unadjusted Cox model, FOLFIRI plus HAIC was associated with improved PFS (HR = 0.62, 95% CI, 0.43–0.89; p = 0.009). Median OS was numerically longer in the FOLFIRI plus HAIC group than in the FOLFOXIRI group (19.5 vs. 17.9 months), but the difference was not statistically significant (log-rank p = 0.150; Cox HR = 0.93, 95% CI, 0.61–1.42; p = 0.739). The R0 resection rate was higher in the FOLFIRI plus HAIC group [14/65 (21.5%) vs. 6/67 (9.0%); p = 0.044]. Grade 3/4 adverse events were comparable between groups [20/65 (30.8%) vs. 26/67 (38.8%); p = 0.333], while grade 3/4 neutropenia showed a borderline lower incidence in the FOLFIRI plus HAIC group [12/65 (18.5%) vs. 19/67 (28.4%); p = 0.051]. HAIC-related complications were infrequent and manageable.

**Conclusions:**

FOLFIRI plus HAIC was associated with improved disease control, prolonged PFS, and a higher R0 resection rate compared with FOLFOXIRI. The regimen showed a manageable safety profile, with infrequent HAIC-related complications. Given the retrospective and non-randomized design, these findings should be interpreted cautiously and validated in prospective randomized studies.

## Introduction

Colorectal cancer (CRC) continues to rank among the most prevalent malignancies globally, contributing significantly to cancer-related morbidity and mortality. It is estimated that over 1.8 million new cases of colorectal cancer are diagnosed annually, with approximately 880,000 deaths (1). The incidence of CRC varies significantly by geographical region, with the highest rates observed in Western countries. Early detection and surgical intervention can lead to favorable outcomes for patients with localized disease. However, in approximately 20-25% of patients at the time of diagnosis, the cancer has already metastasized to distant organs, with the liver being the most common site of metastasis (2). In cases of hepatic metastases, the prognosis remains unfavorable, with systemic chemotherapy typically yielding a median overall survival (OS) of around 24–36 months (3).

The liver is a critical organ for the metabolism and clearance of drugs, making liver metastasis a particularly challenging issue in oncology (4). Liver-dominant metastatic colorectal cancer (CRLM), in which the liver is the primary site of metastatic disease, poses significant therapeutic challenges due to the limited effectiveness of systemic chemotherapy (5). The five-year survival rate for patients with unresectable liver metastases remains low, despite advances in chemotherapy, immunotherapy, and locoregional therapies. Thus, the development of more effective treatment regimens is a priority (6).

Management of metastatic colorectal cancer (mCRC) commonly involves systemic chemotherapy using regimens like FOLFOX and FOLFIRI, both proven effective in reducing systemic tumor burden (7, 8). However, these regimens are not particularly effective against liver-dominant metastasis, especially in patients with extensive liver involvement. FOLFOXIRI, a triplet chemotherapy regimen that combines oxaliplatin, irinotecan, and 5-fluorouracil (5-FU), has been developed to improve treatment outcomes for patients with extensive metastases, including liver metastasis. This regimen has shown improved response rates and survival outcomes compared to FOLFOX and FOLFIRI alone, particularly in patients with a high burden of disease. However, the FOLFOXIRI combination is linked to increased hematological adverse events, particularly neutropenia, potentially restricting its clinical applicability (9).

To overcome the limitations of systemic therapy, hepatic arterial infusion chemotherapy (HAIC) has gained attention as an innovative locoregional strategy for treating liver-dominant metastatic colorectal cancer (10). HAIC involves the administration of high doses of chemotherapy directly into the hepatic artery, providing increased drug concentrations to liver metastases while minimizing systemic exposure and side effects. Preliminary studies have shown that HAIC can enhance the efficacy of systemic chemotherapy regimens, particularly for liver metastases, by delivering higher local drug concentrations directly to the tumor (11, 12). The combination of systemic chemotherapy and HAIC has demonstrated improved outcomes in patients with liver-dominant metastatic colorectal cancer, leading to better tumor control and prolonged survival.

In this study, we compared FOLFOXIRI (a triplet chemotherapy regimen) with FOLFIRI + HAIC (a dual chemotherapy regimen combined with hepatic arterial infusion chemotherapy) in patients with unresectable liver metastases from colorectal cancer. The aim was to determine whether FOLFIRI + HAIC would provide superior treatment outcomes in terms of progression-free survival (PFS), overall survival (OS), and adverse event (AE) profiles, compared to FOLFOXIRI alone.

## Methods

### Study design and patient population

This was a single-center retrospective real-world cohort study conducted at the Department of Medical Oncology, The Affiliated Hospital of YiChun University. Medical records of patients diagnosed with unresectable colorectal liver metastases between January 2019 and December 2023 were retrospectively reviewed. A total of 132 eligible patients were included and divided into two treatment groups according to the first-line treatment regimen they received: FOLFOXIRI and FOLFIRI plus HAIC. The study was approved by the Ethics Committee of The Affiliated Hospital of Yichun University(approval No. YX-AF-SW-01-1.0). Given the retrospective nature of the study, the requirement for written informed consent was waived by the Ethics Committee, and all patient data were anonymized before analysis.

### Treatment allocation

Treatment allocation was not randomized. In this real-world cohort, the selection of FOLFOXIRI or FOLFIRI combined with HAIC reflected routine clinical decision-making at our institution. Treatment decisions were made by the treating oncologists after multidisciplinary assessment, taking into account liver tumor burden, technical feasibility of HAIC, expected tolerance to intensive systemic chemotherapy, molecular profile, patient preference, and availability of interventional radiology support.

Patients assigned to the FOLFOXIRI group generally received triplet systemic chemotherapy when intensive systemic disease control was considered appropriate and when the patient was judged able to tolerate oxaliplatin- and irinotecan-containing systemic therapy. Patients treated with FOLFIRI plus HAIC were generally those with liver-dominant disease for whom improved intrahepatic control was regarded as a major therapeutic objective and in whom hepatic arterial infusion was technically feasible.

Treatment selection may also have been influenced by the increasing availability and clinical experience with HAIC during the later part of the study period. Therefore, treatment period was reviewed as a potential source of allocation bias. Baseline characteristics were compared between the two groups, and multivariable adjustment was performed to reduce the influence of measured confounding factors.

### Inclusion criteria

Patients eligible for this study were:1)Histologically confirmed colorectal cancer (CRC) with liver metastasis, ensuring all participants had a verified diagnosis;2)Presence of measurable hepatic metastases based on RECIST guidelines to facilitate objective assessment of therapeutic response;3)ECOG performance status of 0-2, indicating the patient’s ability to tolerate chemotherapy without significant impairment to daily activities;4)Preserved vital organ functions, including acceptable hepatic indices (ALT, AST, bilirubin), renal function assessed through serum creatinine and estimated glomerular filtration rate (eGFR), as well as normal hematologic parameters, such as leukocyte count, hemoglobin level, and platelet count.

### Exclusion criteria

Individuals who satisfied any of the exclusion parameters outlined below were not included in this investigation: 1)Presence of extrahepatic metastases, as the study focused specifically on liver-dominant metastatic colorectal cancer; 2)Severe comorbidities, including uncontrolled diabetes, heart failure, or significant cardiovascular disease, as these conditions could interfere with the patient’s ability to tolerate chemotherapy or affect treatment outcomes; 3)Prior treatments with hepatic arterial infusion chemotherapy (HAIC), liver resection, radiofrequency ablation (RFA), or transcatheter arterial chemoembolization (TACE), as these therapies could confound the study results by affecting the liver metastasis response to the current treatment regimen; 4)Prior systemic chemotherapy or targeted therapy for metastatic colorectal cancer, which could alter baseline disease status and affect the evaluation of the treatment being studied.

### Treatment regimens

FOLFOXIRI Group: Patients in this group received systemic chemotherapy with FOLFOXIRI (oxaliplatin 85 mg/m², irinotecan 180 mg/m², leucovorin 400 mg/m², 5-FU bolus 400 mg/m² followed by a 2400 mg/m² continuous infusion over 46 hours) administered every two weeks, combined with a targeted agent (bevacizumab or cetuximab) according to the molecular profile (RAS/BRAF status). Treatment continued until disease progression, unacceptable toxicity, or patient withdrawal.

FOLFIRI + HAIC Group: Patients in this group received systemic chemotherapy with FOLFIRI (irinotecan 180 mg/m², leucovorin 400 mg/m², 5-FU bolus 400 mg/m² followed by a 2400 mg/m² continuous infusion over 46 hours) administered every two weeks, combined with a targeted agent (Bevacizumab or cetuximab) according to the molecular profile (e.g., RAS/BRAF status). In addition, hepatic arterial infusion chemotherapy (HAIC) was performed every four weeks, consisting of oxaliplatin (100–150 mg) combined with 5-FU and leucovorin administered via the hepatic artery.

HAIC was performed by experienced interventional radiologists through femoral or radial arterial access. After angiographic evaluation of hepatic arterial anatomy, the catheter tip was positioned in the proper hepatic artery or tumor-feeding branch. Catheter position, arterial patency, and procedure-related complications were monitored during each HAIC cycle.

### Endpoints

The main outcome measure was progression-free survival (PFS), defined as the interval from the beginning of therapy to radiologic evidence of disease advancement or death from any cause. Secondary outcome measures included overall survival (OS), and objective response rate (ORR), which was calculated as the proportion of patients achieving either a complete response (CR) or a partial response (PR) per RECIST guidelines. The disease control rate (DCR) encompassed CR, PR, and stable disease (SD). Adverse events (AEs) were assessed according to the Common Terminology Criteria for Adverse Events (CTCAE) version 5.0, with a specific focus on Grade 3 and 4 toxicities.

### Statistical analysis

Descriptive analyses were performed to summarize baseline characteristics such as age, sex, ECOG performance score, and primary tumor location. Continuous data were expressed as mean ± standard deviation (SD), and categorical variables were presented as percentages. Comparisons of continuous data between groups were conducted using the Student’s t-test, while categorical data were compared using the chi-square test or Fisher’s exact test where appropriate. Survival outcomes, including PFS and OS, were estimated using the Kaplan–Meier method, and differences were assessed using the log-rank test. The Cox proportional hazards regression model was applied to calculate hazard ratios (HRs) for survival outcomes. A p-value < 0.05 was considered statistically significant.

Because treatment allocation was not randomized, multivariable Cox proportional hazards regression was performed to adjust for potential confounders, including age, sex, ECOG performance status, primary tumor site, RAS and BRAF status, liver tumor burden-related variables, baseline CEA level, tumor sidedness, metastasis timing, primary tumor resection status, bilobar liver involvement, and targeted therapy type. Because targeted agents may influence ORR and PFS, stratified analyses were performed according to targeted therapy type, and logistic regression and Cox regression were used to adjust for targeted therapy type, RAS status, and BRAF status.

## Results

### Patient demographics and baseline characteristics

A total of 132 eligible patients were included, with 67 patients in the FOLFOXIRI group and 65 patients in the FOLFIRI plus HAIC group. Baseline demographic, molecular, and liver tumor burden-related characteristics are summarized in **Table 1**. The two groups were generally comparable in age, sex, ECOG performance status, primary tumor location, RAS status, BRAF status, number of liver metastases, largest liver lesion size, baseline CEA level, extent of hepatic involvement, bilobar liver metastases, metastasis timing, and primary tumor status.

**Table 1:** Baseline characteristics of patients.

Variable	FOLFOXIRI (n=67)	FOLFIRI+HAIC (n=65)	p value	Test/statistic
Age, years	58.1 ± 8.7	58.1 ± 8.3	0.990	t=-0.01
Sex, n (%)			0.303	χ²=1.06
Male	41 (61.2%)	34 (52.3%)		
Female	26 (38.8%)	31 (47.7%)		
ECOG performance status, n (%)			0.668	χ²=0.81
0	27 (40.3%)	24 (36.9%)		
1	34 (50.7%)	32 (49.2%)		
2	6 (9.0%)	9 (13.8%)		
Primary tumor location, n (%)			0.881	χ²=0.02
Rectum	28 (41.8%)	28 (43.1%)		
Colon	39 (58.2%)	37 (56.9%)		
RAS status, n (%)			0.726	χ²=0.12
Mutant	34 (50.7%)	31 (47.7%)		
Wild-type	33 (49.3%)	34 (52.3%)		
BRAF status, n (%)			0.956	χ²=0.00
Mutant	6 (9.0%)	6 (9.2%)		
Wild-type	61 (91.0%)	59 (90.8%)		
Number of liver metastases, median (IQR)	5.0 (3.0–6.5)	5.0 (2.0–7.0)	0.803	U=2232
Largest liver lesion size, cm, mean ± SD	4.9 ± 1.5	4.8 ± 1.8	0.686	t=0.41
Baseline CEA, ng/mL, median (IQR)	87.8 (46.8–154.0)	62.1 (39.6–103.1)	0.094	U=2546
Extent of hepatic involvement, n (%)			0.847	χ²=0.33
<25%	33 (49.3%)	30 (46.2%)		
25–50%	25 (37.3%)	24 (36.9%)		
>50%	9 (13.4%)	11 (16.9%)		
Liver lobar involvement, n (%)			0.406	χ²=1.80
Left lobe	4 (6.0%)	6 (9.2%)		
Right lobe	3 (4.5%)	6 (9.2%)		
Bilobar	60 (89.6%)	53 (81.5%)		
Bilobar liver metastases, n (%)			0.190	χ²=1.72
Yes	60 (89.6%)	53 (81.5%)		
No	7 (10.4%)	12 (18.5%)		
Metastasis timing, n (%)			0.622	χ²=0.24
Synchronous	47 (70.1%)	43 (66.2%)		
Metachronous	20 (29.9%)	22 (33.8%)		
Primary tumor status, n (%)			0.302	χ²=1.06
In situ	40 (59.7%)	33 (50.8%)		
Resected	27 (40.3%)	32 (49.2%)		
Primary tumor resected, n (%)			0.302	χ²=1.06
Yes	27 (40.3%)	32 (49.2%)		
No	40 (59.7%)	33 (50.8%)		
Tumor sidedness, n (%)			0.019	χ²=5.47
Left-sided	60 (89.6%)	48 (73.8%)		
Right-sided	7 (10.4%)	17 (26.2%)		

The median number of liver metastases was 5.0 in both groups [5.0 (IQR, 3.0–6.5) vs. 5.0 (IQR, 2.0–7.0); p = 0.803], and the largest liver lesion size was similar between groups (4.9 ± 1.5 cm vs. 4.8 ± 1.8 cm; p = 0.686). Baseline CEA levels were not significantly different [87.8 (IQR, 46.8–154.0) vs. 62.1 (IQR, 39.6–103.1) ng/mL; p = 0.094]. Tumor sidedness differed between groups, with a higher proportion of right-sided tumors in the FOLFIRI plus HAIC group [17/65 (26.2%) vs. 7/67 (10.4%); p = 0.019], and was therefore included as a covariate in adjusted analyses.

### Conversion surgery and local treatment outcomes

Conversion surgery and local treatment outcomes are summarized in **Table 2**. A higher proportion of patients in the FOLFIRI plus HAIC group were converted to resectable disease compared with those in the FOLFOXIRI group [17/65 (26.2%) vs. 9/67 (13.4%)], although the difference did not reach statistical significance (p = 0.066). The proportion of patients who underwent liver resection showed the same distribution [17/65 (26.2%) vs. 9/67 (13.4%); p = 0.066].

**Table 2:** Conversion surgery and local treatment outcomes.

Outcome	FOLFOXIRI (n=67)	FOLFIRI+HAIC (n=65)	p value	Test/statistic
Converted to resectable, n (%)	9 (13.4%)	17 (26.2%)	0.066	χ² = 3.38
Liver resection performed, n (%)	9 (13.4%)	17 (26.2%)	0.066	χ² = 3.38
R0 resection among all patients, n (%)	6 (9.0%)	14 (21.5%)	0.044	χ² = 4.06
Resection margin status among resected patients, n/N (%)			0.127	χ² = 4.13
R0	6/9 (66.7%)	14/17 (82.4%)		
R1	3/9 (33.3%)	1/17 (5.9%)		
R2	0/9 (0.0%)	2/17 (11.8%)		
Primary tumor resection performed, n (%)	30 (44.8%)	37 (56.9%)	0.163	χ² = 1.95
Ablation performed, n (%)	11 (16.4%)	9 (13.8%)	0.680	χ² = 0.17
SBRT performed, n (%)	2 (3.0%)	3 (4.6%)	0.678	Fisher’s exact test
Other local therapy, n (%)	22 (32.8%)	29 (44.6%)	0.165	χ² = 1.93

The R0 resection rate among all patients was significantly higher in the FOLFIRI plus HAIC group than in the FOLFOXIRI group [14/65 (21.5%) vs. 6/67 (9.0%); p = 0.044]. Among patients who underwent liver resection, R0 resection was achieved in 14 of 17 patients (82.4%) in the FOLFIRI plus HAIC group and in 6 of 9 patients (66.7%) in the FOLFOXIRI group. The overall distribution of resection margin status did not differ significantly between the two groups (p = 0.127).

No significant between-group differences were observed in the use of ablation [9/65 (13.8%) vs. 11/67 (16.4%); p = 0.680], SBRT [3/65 (4.6%) vs. 2/67 (3.0%); p = 0.678], or other local therapies [29/65 (44.6%) vs. 22/67 (32.8%); p = 0.165].

### Efficacy outcomes

Tumor response and survival outcomes are summarized in **Table 3**. The ORR was numerically higher in the FOLFIRI plus HAIC group than in the FOLFOXIRI group [37/65 (56.9%) vs. 30/67 (44.8%)], but the difference was not statistically significant (p = 0.163). CR and PR rates were also numerically higher in the FOLFIRI plus HAIC group, but neither reached statistical significance. In contrast, PD occurred significantly less frequently in the FOLFIRI plus HAIC group [7/65 (10.8%) vs. 21/67 (31.3%); p = 0.004], and the DCR was significantly higher [58/65 (89.2%) vs. 46/67 (68.7%); p = 0.004].

**Table 3:** Treatment response and survival outcomes.

Outcome	FOLFOXIRI (n=67)	FOLFIRI+HAIC (n=65)	HR (95% CI)	p value	χ²
Objective response rate (ORR), n (%)	30 (44.8%)	37 (56.9%)	—	0.163	χ²=1.95
Complete response (CR), n (%)	6 (9.0%)	10 (15.4%)	—	0.258	χ²=1.28
Partial response (PR), n (%)	24 (35.8%)	27 (41.5%)	—	0.500	χ²=0.45
Stable disease (SD), n (%)	16 (23.9%)	21 (32.3%)	—	0.281	χ²=1.16
Progressive disease (PD), n (%)	21 (31.3%)	7 (10.8%)	—	0.004	χ²=8.36
Disease control rate (DCR), n (%)	46 (68.7%)	58 (89.2%)	—	0.004	χ²=8.36
Median PFS, months	8.2	10.3	0.62(0.43–0.89)	0.038	—
Median OS, months	17.9	19.5	0.93 (0.61–1.42)	0.15	—

For survival outcomes, p values were calculated using the log-rank test, and HRs were estimated using unadjusted Cox proportional hazards regression. HR, hazard ratio; CI, confidence interval; PFS, progression-free survival; OS, overall survival.

**Figure 1:**
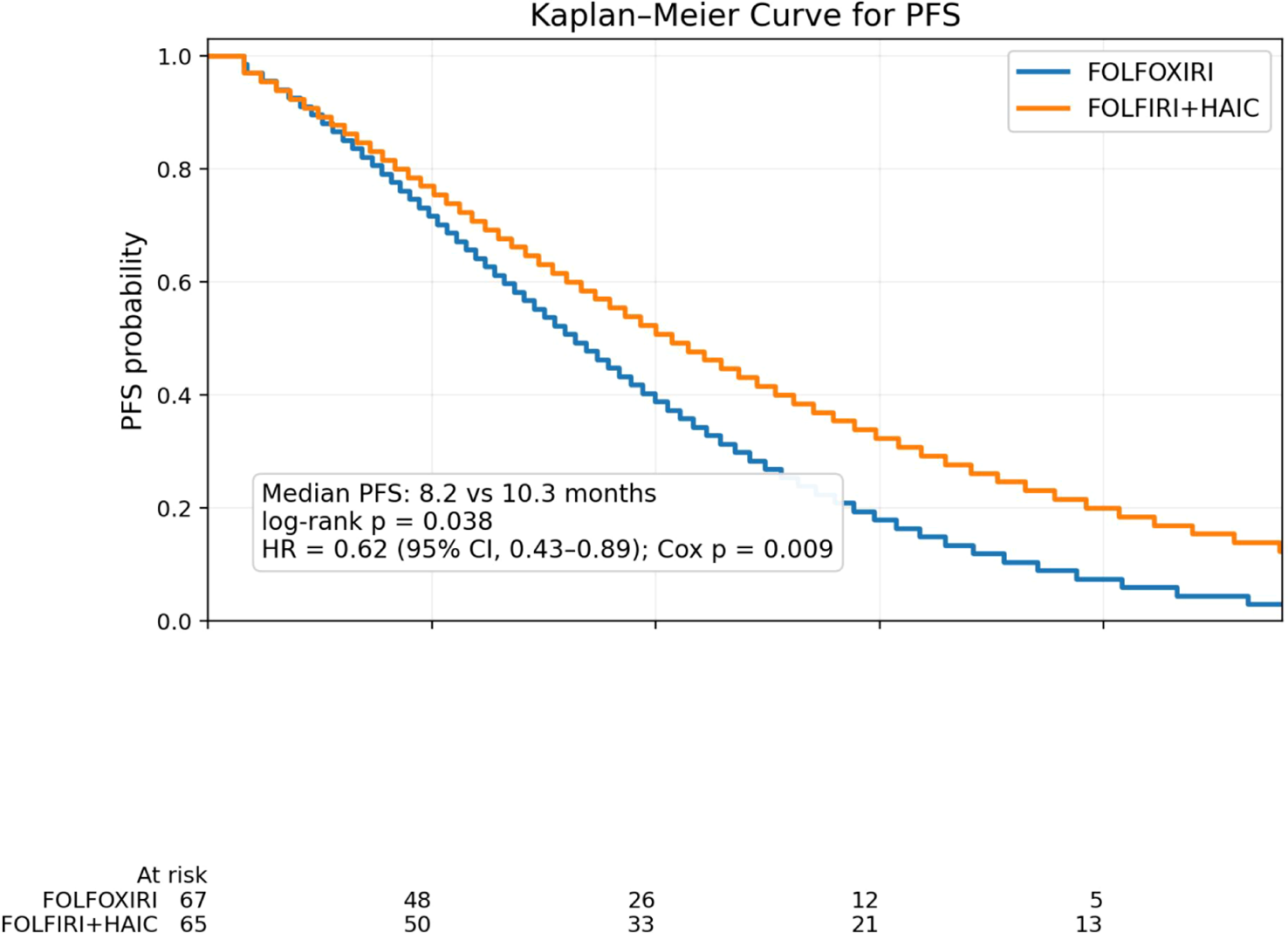
Kaplan–Meier survival curves for progression-free survival.

**Figure 2:**
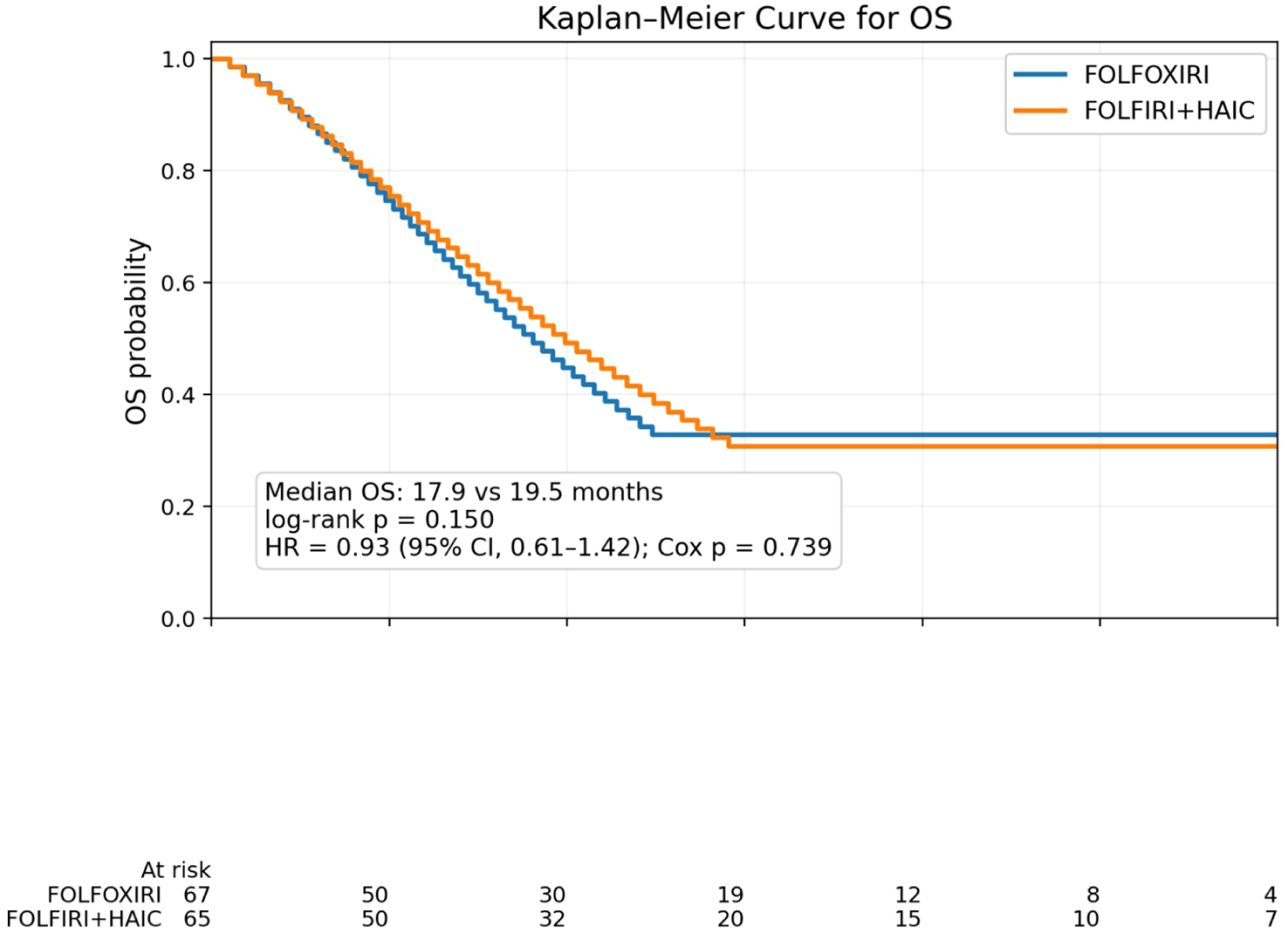
Kaplan–Meier survival curves for overall survival.

Kaplan–Meier survival curves for PFS and OS are shown in **Figure 1** and **2**, respectively. The median follow-up time, estimated using the reverse Kaplan–Meier method, was 31.1 months in the FOLFOXIRI group and 32.7 months in the FOLFIRI plus HAIC group. As shown in **Figure 1**, FOLFIRI plus HAIC significantly prolonged PFS compared with FOLFOXIRI, with a median PFS of 10.3 versus 8.2 months (log-rank p = 0.038). In the unadjusted Cox model, FOLFIRI plus HAIC was associated with improved PFS (HR = 0.62, 95% CI, 0.43–0.89; p = 0.009). As shown in **Figure 2**, median OS was numerically longer in the FOLFIRI plus HAIC group than in the FOLFOXIRI group (19.5 vs. 17.9 months), but the difference was not statistically significant (log-rank p = 0.150; Cox HR = 0.93, 95% CI, 0.61–1.42; p = 0.739). Numbers at risk over time are provided below each Kaplan–Meier curve.

Given the non-randomized design of this study, Cox proportional hazards regression was performed to adjust for potential confounding factors, and the results are summarized in **Table 4**. In the unadjusted Cox model, FOLFIRI plus HAIC was associated with improved PFS compared with FOLFOXIRI (HR = 0.62, 95% CI, 0.43–0.89; p = 0.009). This association remained significant after multivariable adjustment (adjusted HR = 0.62, 95% CI, 0.41–0.93; p = 0.021). For OS, no significant survival benefit was observed in either the unadjusted model (HR = 0.93, 95% CI, 0.61–1.42; p = 0.739) or the multivariable model (adjusted HR = 0.90, 95% CI, 0.54–1.49; p = 0.676).

**Table 4:** Unadjusted and multivariable Cox proportional hazards regression analyses for progression-free survival and overall survival.

Endpoint	Analysis model	HR for FOLFIRI+HAIC vs FOLFOXIRI	Lower 95% CI	Upper 95% CI	p value
PFS	Unadjusted Cox model	0.62	0.43	0.89	0.009
PFS	Multivariable Cox model	0.620	0.410	0.93	0.021
OS	Unadjusted Cox model	0.93	0.61	1.42	0.739
OS	Multivariable Cox model	0.90	0.540	1.49	0.676

### Sensitivity and stratified analyses according to targeted therapy

Targeted therapy distribution was further evaluated according to RAS and BRAF status. As shown in **Table 5**, among patients with RAS wild-type tumors, the distribution of anti-VEGF and anti-EGFR therapy was comparable between the FOLFOXIRI and FOLFIRI plus HAIC groups. Anti-VEGF therapy was used in 21/33 patients (63.6%) in the FOLFOXIRI group and 22/34 patients (64.7%) in the FOLFIRI plus HAIC group, while anti-EGFR therapy was used in 12/33 (36.4%) and 12/34 (35.3%) patients, respectively (p = 0.927). All patients with RAS-mutant tumors received anti-VEGF therapy. As shown in **Table 6**, a similar balance was observed according to BRAF status. Among patients with BRAF wild-type tumors, anti-VEGF therapy was used in 49/61 patients (80.3%) in the FOLFOXIRI group and 47/59 patients (79.7%) in the FOLFIRI plus HAIC group, whereas anti-EGFR therapy was used in 12/61 (19.7%) and 12/59 (20.3%) patients, respectively (p = 0.927). All patients with BRAF-mutant tumors received anti-VEGF therapy.

**Table 5:** Distribution of targeted therapy according to RAS status.

RAS status	Treatment group	n	Anti-VEGF, n (%)	Anti-EGFR, n (%)	p value
RAS wild-type	FOLFOXIRI	33	21 (63.6)	12 (36.4)	0.927
RAS wild-type	FOLFIRI+HAIC	34	22 (64.7)	12 (35.3)	
RAS mutant	FOLFOXIRI	34	34 (100.0)	0 (0.0)	NA
RAS mutant	FOLFIRI+HAIC	31	31 (100.0)	0 (0.0)	

**Table 6:** Distribution of targeted therapy according to BRAF status.

BRAF status	Treatment group	n	Anti-VEGF, n (%)	Anti-EGFR, n (%)	p value
BRAF wild-type	FOLFOXIRI	61	49 (80.3)	12 (19.7)	0.927
BRAF wild-type	FOLFIRI+HAIC	59	47 (79.7)	12 (20.3)	
BRAF mutant	FOLFOXIRI	6	6 (100.0)	0 (0.0)	NA
BRAF mutant	FOLFIRI+HAIC	6	6 (100.0)	0 (0.0)	

As shown in **Table 7**, stratified analysis according to targeted therapy type showed a numerically higher ORR in the FOLFIRI plus HAIC group in both the anti-VEGF subgroup [30/53 (56.6%) vs. 27/55 (49.1%); p = 0.434] and the anti-EGFR subgroup [7/12 (58.3%) vs. 3/12 (25.0%); p = 0.214]. The total ORR across targeted therapy subgroups was consistent with the corrected overall ORR analysis [37/65 (56.9%) vs. 30/67 (44.8%)]. As shown in **Table 8**, after adjustment for targeted therapy type, RAS status, and BRAF status, FOLFIRI plus HAIC remained associated with a numerically higher ORR, but the association was not statistically significant (adjusted OR = 1.68, 95% CI, 0.83–3.38; p = 0.147).

**Table 7:** Stratified analysis of ORR according to targeted therapy type.

Targeted therapy type	FOLFOXIRI	FOLFIRI+HAIC	p value	Test/statistic
anti-VEGF	27/55 (49.1%)	30/53 (56.6%)	0.434	χ²=0.61
anti-EGFR	3/12 (25.0%)	7/12 (58.3%)	0.214	Fisher’s exact test

**Table 8:** Adjusted logistic regression for ORR.

Outcome	Adjusted OR for FOLFIRI+HAIC vs FOLFOXIRI	95% CI	p value
ORR	1.68	0.83–3.38	0.147

As shown in **Table 9**, PFS was significantly longer in the FOLFIRI plus HAIC group in the anti-VEGF subgroup, with a median PFS of 10.4 months compared with 7.9 months in the FOLFOXIRI group (HR = 0.60, 95% CI, 0.41–0.89; p = 0.012). In the anti-EGFR subgroup, the median PFS was 8.7 months in the FOLFIRI plus HAIC group and 12.0 months in the FOLFOXIRI group, with no statistically significant difference (HR = 0.71, 95% CI, 0.29–1.69; p = 0.433). As shown in **Table 10**, Cox models further confirmed that the PFS benefit associated with FOLFIRI plus HAIC remained significant after adjustment for targeted therapy type alone (HR = 0.61, 95% CI, 0.42–0.87; p = 0.007) and after additional adjustment for RAS and BRAF status (HR = 0.60, 95% CI, 0.42–0.87; p = 0.007).

**Table 9:** Stratified analysis of PFS according to targeted therapy type.

Targeted therapy type	Median PFS, FOLFOXIRI	Median PFS, FOLFIRI+HAIC	HR	95% CI	p value
anti-VEGF	7.9 months	10.4 months	0.60	0.41–0.89	0.012
anti-EGFR	12.0 months	8.7 months	0.71	0.29–1.69	0.433

**Table 10:** Cox models for PFS adjusted for targeted therapy type, RAS, and BRAF status.

Model	HR for FOLFIRI+HAIC vs FOLFOXIRI	95% CI	p value
Unadjusted Cox	0.62	0.43–0.89	0.009
Adjusted for targeted therapy type	0.61	0.42–0.87	0.007
Adjusted for targeted therapy type, RAS and BRAF status	0.60	0.42–0.87	0.007

### Safety profile

**Table 11** summarizes grade 3/4 adverse events. The overall incidence of grade 3/4 adverse events was comparable between the FOLFOXIRI and FOLFIRI plus HAIC groups [26/67 (38.8%) vs. 20/65 (30.8%); p = 0.333]. Grade 3/4 neutropenia was more frequent in the FOLFOXIRI group [19/67 (28.4%) vs. 12/65 (18.5%)], although the difference was only borderline significant (p = 0.051). Grade 3/4 diarrhea [9/67 (13.4%) vs. 6/65 (9.2%); p = 0.23] and ALT/AST elevation [6/67 (9.0%) vs. 9/65 (13.8%); p = 0.21] were not significantly different between groups. Other grade 3/4 toxicities were uncommon, and no significant between-group differences were observed.

**Table 11:** Adverse events (AEs).

Adverse event	FOLFOXIRI (n=67)	FOLFIRI+HAIC (n=65)	p value	Test/statistic
Neutropenia (G3/4)	19 (28.4%)	12 (18.5%)	0.051	3.89
Diarrhea (G3/4)	9 (13.4%)	6 (9.2%)	0.23	1.42
ALT/AST Elevation (G3/4)	6 (9.0%)	9 (13.8%)	0.21	1.56
Fatigue (G3/4)	4 (6.0%)	4 (6.2%)	0.85	0.02
Nausea/Vomiting (G3/4)	4 (6.0%)	3 (4.6%)	0.73	0.17
Leukopenia (G3/4), n (%)	7 (10.4%)	3 (4.6%)	0.325	Fisher
Febrile neutropenia (G3/4), n (%)	1 (1.5%)	0 (0.0%)	1.000	Fisher
Anemia (G3/4), n (%)	2 (3.0%)	1 (1.5%)	1.000	Fisher
Thrombocytopenia (G3/4), n (%)	1 (1.5%)	1 (1.5%)	1.000	Fisher
Peripheral neuropathy (G3/4), n (%)	0 (0.0%)	0 (0.0%)	NA	NA
Bilirubin increase (G3/4), n (%)	1 (1.5%)	1 (1.5%)	1.000	Fisher
Infection (G3/4), n (%)	5 (7.5%)	2 (3.1%)	0.441	Fisher

### HAIC technical details and procedure-related complications.

HAIC-related parameters and complications are summarized in **Table 12**. Technical success was achieved in all 65 patients (100.0%) in the FOLFIRI plus HAIC group. HAIC interruption occurred in 5 patients (7.7%), and 3 patients (4.6%) discontinued HAIC because of procedure-related complications. The arterial access route was femoral in 28 patients (43.1%) and radial in 37 patients (56.9%). The catheter tip was positioned in the proper hepatic artery or tumor-feeding branch in all patients. The median number of HAIC cycles was 4.0 (IQR, 3.0–6.0), and the median infusion duration was 46.0 hours.

**Table 12:** HAIC technical parameters and procedure-related complications.

HAIC-related parameter/complication	FOLFIRI+HAIC (n=65)
HAIC technical success	65 (100.0%)
HAIC interruption	5 (7.7%)
HAIC discontinuation due to complication	3 (4.6%)
Arterial access route	
Femoral artery	28 (43.1%)
Radial artery	37 (56.9%)
Catheter tip position	
Proper hepatic artery or tumor-feeding branch	65 (100.0%)
Port system used	
No	55 (84.6%)
Yes	10 (15.4%)
HAIC cycles, median (IQR)	4.0 (3.0–6.0)
HAIC infusion duration, hours, median (IQR)	46.0 (46.0–46.0)
Catheter occlusion	1 (1.5%)
Catheter displacement	3 (4.6%)
Catheter infection	1 (1.5%)
Catheter-related thrombosis	1 (1.5%)
Hepatic artery spasm	6 (9.2%)
Hepatic artery stenosis	1 (1.5%)
Hepatic artery dissection	0 (0.0%)
Cholangitis	1 (1.5%)
Biliary injury	2 (3.1%)
Puncture-site complication	2 (3.1%)

Procedure-related complications were uncommon. Catheter displacement occurred in 3 patients (4.6%), while catheter occlusion, catheter infection, and catheter-related thrombosis each occurred in 1 patient (1.5%). Hepatic artery spasm was observed in 6 patients (9.2%), hepatic artery stenosis in 1 patient (1.5%), and no hepatic artery dissection was reported. Biliary complications included cholangitis in 1 patient (1.5%) and biliary injury in 2 patients (3.1%). Puncture-site complications occurred in 2 patients (3.1%).

### Treatment exposure and subsequent therapy

Treatment exposure and subsequent therapies are summarized in **Table 13**. Compared with the FOLFOXIRI group, patients in the FOLFIRI plus HAIC group received more systemic chemotherapy cycles [9 (IQR, 7–11) vs. 8 (IQR, 7–9); p < 0.001] and had a longer duration of systemic therapy [18.9 weeks (IQR, 14.7–23.1) vs. 16.8 weeks (IQR, 14.7–18.9); p < 0.001]. Relative dose intensity was similar between groups [0.9 (IQR, 0.8–0.9) in both groups], although the difference reached statistical significance (p = 0.048). The median number of targeted therapy cycles was comparable between groups [9 (IQR, 7–12) vs. 9 (IQR, 7–11); p = 0.093]. In the FOLFIRI plus HAIC group, the median number of HAIC cycles was 4 (IQR, 3–6).

**Table 13:** Subsequent local therapy after first-line treatment.

Variable	FOLFOXIRI (n=67)	FOLFIRI+HAIC (n=65)	p value	Test/statistic
Systemic chemotherapy cycles, median (IQR)	8 (7–9)	9 (7–11)	<0.001	U=1417
Duration of systemic therapy, weeks, median (IQR)	16.8 (14.7–18.9)	18.9 (14.7–23.1)	<0.001	U=1431
Relative dose intensity, median (IQR)	0.9 (0.8–0.9)	0.9 (0.8–0.9)	0.048	U=1742
Targeted therapy cycles, median (IQR)	9 (7–11)	9 (7–12)	0.093	U=1810
HAIC cycles, median (IQR)	NA	4 (3–6)	NA	Descriptive only
Dose reduction, n (%)			0.913	χ²=0.01
Yes	16 (23.9%)	15 (23.1%)		
No	51 (76.1%)	50 (76.9%)		
Treatment delay, n (%)			0.275	χ²=1.19
Yes	20 (29.9%)	14 (21.5%)		
No	47 (70.1%)	51 (78.5%)		
Treatment discontinuation due to AE, n (%)			0.793	χ²=0.07
Yes	6 (9.0%)	5 (7.7%)		
No	61 (91.0%)	60 (92.3%)		
Second-line therapy received, n (%)			0.925	χ²=0.01
Yes	52 (77.6%)	50 (76.9%)		
No	15 (22.4%)	15 (23.1%)		
Third-line therapy received, n (%)			0.520	χ²=0.41
Yes	22 (32.8%)	18 (27.7%)		
No	45 (67.2%)	47 (72.3%)		
Immunotherapy received, n (%)			1.000	Fisher’s exact test
Yes	2 (3.0%)	2 (3.1%)		
No	65 (97.0%)	63 (96.9%)		
Subsequent local therapy, n (%)			0.096	χ²=2.77
Yes	5 (7.5%)	11 (16.9%)		
No	62 (92.5%)	54 (83.1%)		
Reason for first-line treatment discontinuation, n (%)			0.264	χ²=5.24
Disease progression	31 (46.3%)	29 (44.6%)		
Completed planned therapy	14 (20.9%)	17 (26.2%)		
Conversion surgery	5 (7.5%)	9 (13.8%)		
Toxicity	10 (14.9%)	3 (4.6%)		
Patient preference	7 (10.4%)	7 (10.8%)		
Second-line regimen, n (%)			<0.001	χ²=98.07
FOLFOX	15 (22.4%)	0 (0.0%)		
FOLFIRI	21 (31.3%)	0 (0.0%)		
FOLFOX/FOLFIRI switch	1 (1.5%)	50 (76.9%)		
TAS-102	15 (22.4%)	0 (0.0%)		
None/not received	15 (22.4%)	15 (23.1%)		
Type of subsequent local therapy, n (%)			0.390	χ²=4.12
Surgery	1 (1.5%)	4 (6.2%)		
Ablation	1 (1.5%)	4 (6.2%)		
TACE	2 (3.0%)	2 (3.1%)		
SBRT	1 (1.5%)	1 (1.5%)		
None	62 (92.5%)	54 (83.1%)		

Dose reduction, treatment delay, and treatment discontinuation due to adverse events were not significantly different between the two groups. Dose reduction occurred in 16 patients (23.9%) in the FOLFOXIRI group and 15 patients (23.1%) in the FOLFIRI plus HAIC group (p = 0.913). Treatment delay occurred in 20 patients (29.9%) and 14 patients (21.5%), respectively (p = 0.275), while treatment discontinuation due to adverse events occurred in 6 patients (9.0%) and 5 patients (7.7%), respectively (p = 0.793).

Subsequent treatment patterns were also recorded. The proportions of patients receiving second-line therapy [52/67 (77.6%) vs. 50/65 (76.9%); p = 0.925], third-line therapy [22/67 (32.8%) vs. 18/65 (27.7%); p = 0.520], immunotherapy [2/67 (3.0%) vs. 2/65 (3.1%); p = 1.000], and subsequent local therapy [5/67 (7.5%) vs. 11/65 (16.9%); p = 0.096] were not significantly different between groups. The reasons for first-line treatment discontinuation were also comparable (p = 0.264), with disease progression being the most common reason in both groups. However, the distribution of second-line regimens differed significantly between groups (p < 0.001), mainly reflecting treatment sequencing after the initial first-line regimen.

## Discussion

The management of CRLM remains a formidable challenge in oncology due to its unique biological behavior and the liver’s central role in drug metabolism. Although standard chemotherapy regimens such as FOLFOXIRI and FOLFIRI have demonstrated efficacy in controlling systemic disease, their therapeutic impact on liver metastases is often suboptimal. This limitation is particularly evident in patients with a high tumor burden in the liver, where insufficient intrahepatic drug concentration results in inadequate local control (13, 14). Therefore, developing more targeted strategies to enhance locoregional efficacy and prolong survival is of paramount clinical importance.

HAIC delivers cytotoxic agents directly into the hepatic artery, leading to higher local drug concentrations in liver metastases while minimizing systemic exposure due to the liver’s first-pass metabolism (15). HAIC has gained increasing attention for its potential in managing liver metastases, especially when combined with systemic chemotherapy. In our real-world retrospective study, FOLFIRI plus HAIC was associated with improved disease control and prolonged PFS compared with FOLFOXIRI. Although the ORR was numerically higher in the FOLFIRI plus HAIC group, the difference was not statistically significant after correction and adjustment for targeted therapy type, RAS status, and BRAF status, suggesting a potential advantage in delaying disease progression, while the improvement in tumor response should be interpreted cautiously.

Although triplet chemotherapy regimens such as FOLFOXIRI have been associated with higher response rates, they are also linked to increased hematologic toxicities—particularly neutropenia (28.4% in our cohort). In contrast, the FOLFIRI + HAIC combination demonstrated a more favorable safety profile, with a lower incidence of grade 3/4 neutropenia (18.5%, p=0.051), suggesting that HAIC may help mitigate the systemic toxicity frequently observed with more intensive regimens. Moreover, the locoregional enhancement achieved through HAIC may improve drug perfusion in liver metastases and increase chemosensitivity.

Our findings are consistent with previous studies, such as that of Huang’s study (16), which demonstrated that the addition of HAIC to systemic FOLFIRI significantly improved PFS and OS compared to FOLFIRI alone. In alignment with these results, our data suggest that HAIC was associated with prolonged PFS and a numerically higher tumor response, although the ORR advantage was attenuated after adjustment for targeted therapy type, RAS status, and BRAF status. In an era where immunotherapy has not yet substantially reshaped the treatment landscape of metastatic colorectal cancer (mCRC), optimizing chemotherapeutic delivery remains a viable and clinically relevant strategy.

Nevertheless, this study has several limitations. Firstly, as a single-center retrospective analysis, it is inherently subject to selection and information biases, which could affect the generalizability of our findings. In addition, treatment allocation was not randomized and may have been influenced by physician preference, patient clinical characteristics, technical feasibility of HAIC, and temporal changes in institutional practice. Although baseline variables were compared between groups and adjusted analyses were performed to reduce measured confounding, residual unmeasured confounding could not be completely excluded. Secondly, although the sample size was adequate for initial comparison, it may not have been large enough to detect long-term survival benefits or rare adverse events. Although we performed stratified and adjusted analyses according to targeted therapy type, RAS status, and BRAF status, other biologically relevant markers, such as VEGF expression, were not available for all patients. Lastly, the application of HAIC requires interventional radiology support and multidisciplinary collaboration, which may limit its feasibility in resource-limited settings and warrants further cost-effectiveness analysis.

## Conclusion

In conclusion, FOLFIRI plus HAIC was associated with improved disease control and prolonged PFS compared with FOLFOXIRI in patients with unresectable colorectal liver metastases. The ORR was numerically higher but did not reach statistical significance. Given the retrospective and non-randomized design, these findings should be interpreted cautiously and validated in prospective randomized studies.

## Data Availability

The original contributions presented in the study are included in the article/supplementary material. Further inquiries can be directed to the corresponding author.
